# Knowledge of Sleep Disorders Among Physicians at a Tertiary Care Hospital in Qatar: Cross-sectional Study

**DOI:** 10.2196/25606

**Published:** 2021-05-12

**Authors:** Irfan Ul Haq, Mansoor Ali Hameed, Merlin Marry Thomas, Khezar Shahzada Syed, Ahmad Mohammad Mahmoud Othman, Shakeel Ahmed, Abbas Abdallah Alabbas, Mushtaq Ahmad

**Affiliations:** 1 Hamad Medical Corporation Doha Qatar; 2 Weill Cornell Medicine-Qatar Cornell University Doha Qatar

**Keywords:** sleep disordered breathing, obstructive sleep apnea, sleep, physician, physician knowledge, sleep disorder, survey method, attitudes, practice

## Abstract

**Background:**

Sleep disorders constitute a major health problem because of their relatively high and rising prevalence. Several studies worldwide have analyzed health care providers’ knowledge of sleep disorders.

**Objective:**

In this study, we aimed to assess the knowledge of sleep disorders among physicians in Qatar.

**Methods:**

A total of 250 physicians were surveyed regarding their knowledge of sleep medicine by using the validated 30-item Assessment of Sleep Knowledge in Medical Education (ASKME) Survey. The participants included residents, fellows, and consultants in medicine and allied subspecialties. A high score was defined as ≥60% of correctly answered questions, implying the respondent has adequate knowledge of sleep disorders.

**Results:**

Responses were received from 158 of the 250 physicians, with a response rate of 63.2%. This included responses from 34 residents, 74 clinical fellows, and 50 consultants. The overall mean score was 15.53 (SD 4.42), with the highest possible score of 30. Only 57 of 158 (36.1%) respondents were able to answer ≥60% of the questions correctly. No statistically significant difference was found in the scores of participants with regard to their ranks (ie, residents, fellows, or consultants) or years of medical training.

**Conclusions:**

This study demonstrates that health care providers in Qatar have decreased awareness and knowledge about sleep medicine, which may reflect reduced emphasis on sleep disorders during medical school and training. Increasing awareness regarding sleep medicine among nonspecialist physicians will allow early detection and treatment of sleep disorders, thereby reducing the morbidity associated with these disorders.

## Introduction

Sleep disorders are defined as a range of sleep problems, including conditions causing hypersomnia (such as sleep apnea and narcolepsy), parasomnia (restless leg syndrome and sleepwalking), insomnia, and sleep-wake cycle disturbances. All these sleep disorders share a common outcome—nonrestorative sleep [1]. Excessive daytime somnolence (EDS) is a consequence of sleep disorder, which can impact focus, concentration, and memory. Nonrestorative sleep and EDS are related to respiratory, cardiovascular, and neurological problems such as increased reaction time, which in turn can lead to motor vehicle and other serious accidents in situations where alertness is required for safety and critical decision-making [2-4]. Hence, sleep disorders are a major risk to public health. Obstructive sleep apnea (OSA), central sleep apnea, and obesity hypoventilation syndrome are treatable sleep disorders that affect a significant proportion of the population worldwide, with OSA prevalent in 3%-5% of middle-aged men and 2%-5% of women [5]. A population-based regional survey evaluating OSA in Saudi Arabia found similar data, with OSA prevalence reported at 4% and 1.8% among men and women, respectively [6].

Given the impact that sleep disorders have on the health and well-being of a significant portion of society, physicians, regardless of their specialty, will inevitably encounter patients with sleep complaints; they should, therefore, have the knowledge and awareness to diagnose sleep disorders. Unfortunately, despite the common presentation and clinical significance of these conditions, sleep disorders remain underdiagnosed or misdiagnosed and, consequently, untreated [7]. In the 2005 National Sleep Foundation's *Sleep in America* poll, 70% of the respondents reported that their doctor had never asked about their sleep habits or patterns [8].

As a result, sleep disorders and associated modifiable risk factors such as obesity remain unaddressed and continue to progress, leading to the worsening of disordered sleep patterns and their ensuing complications [9].

Limited studies have addressed the knowledge of sleep disorders among practicing health care practitioners in the Middle East and, to the best of our knowledge, no similar studies have been conducted on health care practitioners in the State of Qatar. Therefore, we aimed to address this gap by conducting a survey to assess the knowledge of sleep disorders among physicians working at a tertiary care center in the State of Qatar.

## Methods

### Study Group

We conducted a survey-based study from August 2018 to December 2018. The target population comprised postgraduate medical trainees and health care practitioners. Our study sample included residents in the Internal Medicine program and fellows in allied medical subspecialties, undertraining programs at Hamad Medical Corporation accredited by the Accreditation Council for Graduate Medical Education–International (ACGME-I), and consultants in General Medicine and subspecialties at Hamad General and Heart Hospitals, Qatar.

### Survey

We used the Assessment of Sleep Knowledge in Medical Education (ASKME) Survey, a validated 30-item questionnaire that has been designed as a standardized tool for the assessment of medical education in sleep [10]. The survey assesses five separate areas of sleep knowledge, including (1) basic sleep principles, (2) circadian sleep/wake control, (3) normal sleep architecture, (4) common sleep disorders, and (5) effects of drugs and alcohol on sleep. Possible responses to the survey items are “true,” “false,” and “I don’t know.” Participants were categorized into two groups: (1) a high score group comprising participants with correct scores ≥60% and (2) a low score group comprising participants with correct scores <60%, based on the cut-off pass threshold mark used in the majority of medical schools across the Gulf states.

### Statistical Analysis

Continuous data are presented as means and SD, and categorical data are presented in the text and tables as absolute numbers (n) and percentages (%). One-way analysis of variance (ANOVA) was performed for comparison between more than two groups. A *P* value ≤.05 was significant. Statistical analysis was conducted using Stata Statistical Software (Release 16; StataCorp LLC).

## Results

The survey was administered to 250 participants. Responses were received from 158 participants, with a response rate of 63.2%. These included data collected from 34 residents in internal medicine; 74 clinical fellows training in internal medicine, cardiology, endocrinology, neurology, rheumatology, and nephrology; and 50 consultants in general medicine (Table 1). The majority of respondents (121/158, 76.6%) were male and aged above 30 years (Table 1). The participants’ mean overall score was 15.53 (SD 4.42), with the highest possible score of 30. Only 57 of 158 (36.1%) participants scored ≥60%.

**Table 1:** Demographics of the survey participants (N=158).

Variable		Participants, n (%)
**Age (years)**		
	25-30	37 (23.4)
	>30	121 (76.6)
**Gender**		
	Male	131 (82.9)
	Female	27 (17.1)
**Level of training**		
	Resident	34 (21.5)
	Fellow	74 (46.8)
	Consultant	50 (31.6)
**Year of training**		
	First year	25 (15.8)
	Second year	16 (10.1)
	Third year	32 (20.3)
	Fourth year	11 (7.0)
	More than 4 years	74 (46.8)
**Country of graduation**		
	Pakistan	25 (15.8)
	Jordan	16 (10.1)
	Libya	14 (8.9)
	Syria	12 (7.6)
	Sudan	12 (7.6)
	India	10 (6.3)
	Egypt	8 (5.1)
	Qatar	7 (4.4)
	United Kingdom	4 (2.5)
	Palestine	4 (2.5)
	Ireland	3 (1.9)
	Yemen	3 (1.9)
	United Arab Emirates	2 (1.3)
	Unknown^a^	38 (24.1)

^a^38 respondents did not respond to the question about their country of graduation.

**Figure 1:**
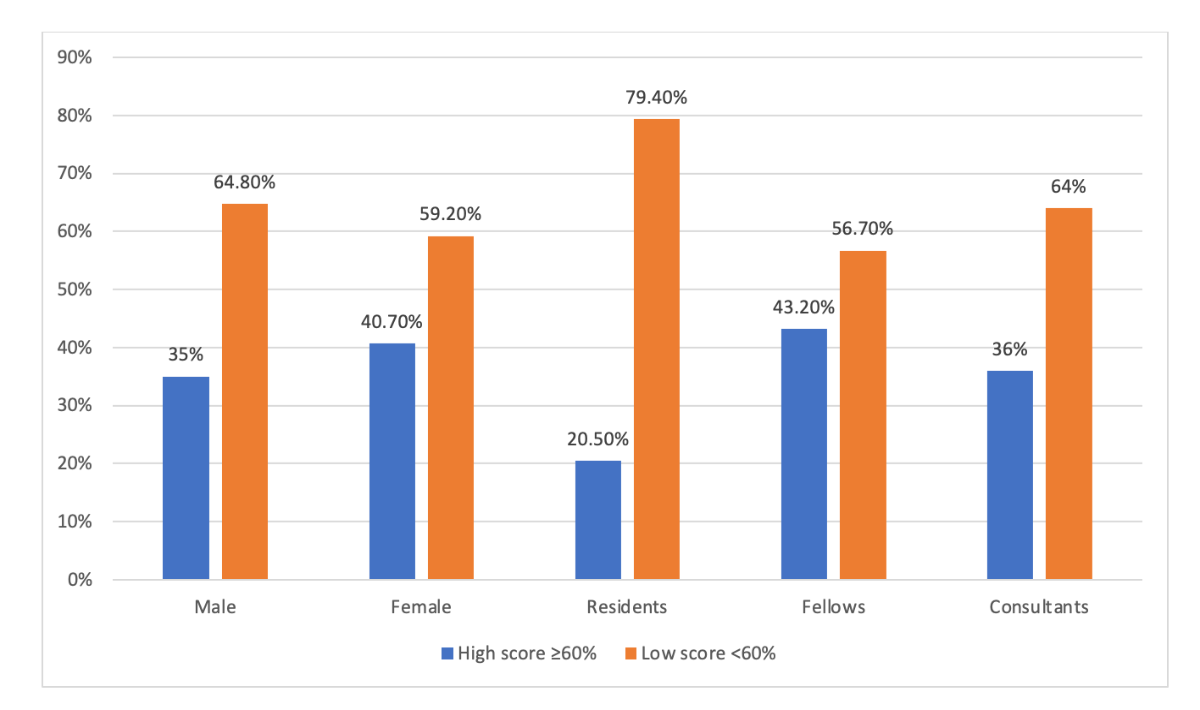
Percentages of high and low scores across gender and different groups of physicians.

Further analysis showed that the high-score group (n=57) comprised 7 (12%) residents, 32 (56%) fellows, and 18 (32%) consultants, whereas the low-score group (n=101) comprised 27 (26.7%) residents, 42 (41.6%) fellows, and 32 (31.7%) consultants (Table 2). Figure 1 shows the percentage of high and low scores across gender and different groups of physicians. No statistically significant difference was found between the scores of respondents in the 25- to 30-year age group and those aged above 30 years. Rank of the physician (ie, residents, fellows, or consultants), year of training among residents and fellows, and country of graduation also did not have a statistically significant effect on the total scores (Table 3).

**Table 2:** Comparison of age, gender, and designation of participants in the high-score group (n=57).

Variable		Participants who scored ≥60%, n (%)		*P* value
**Age (years)**				.62
	25-30 (n=37)	12 (32)		
	>30 (n=121)	45 (37)		
**Gender**				.59
	Male (n=131)	46 (35)		
	Female (n=27)	11 (41)		
**Level of training**				.23
	Residents (n=34)	7 (21)		
	Fellows (n=74)	32 (43)		
	Consultants (n=50)	18 (36)		

**Table 3:** Comparison of the years and level of training and country of graduation of participants in the high-score group (n=57).

Variable				Participants who scored ≥60%, n (%)		*P* value
**Year of training**						.84
	**Resident**					
		First year (n=10)	2 (20)			
		Second year (n=4)	2 (50)			
		Third year (n=16)	2 (13)			
		Fourth year (n=4)	1 (25)			
	**Fellow**					
		First year (n=15)	7 (47)			
		Second year (n=12)	6 (50)			
		Third year (n=16)	8 (50)			
		Fourth year (n=5)	1 (20)			
		More than 4 years (n=26)	10 (38)			
**Country of graduation**						.63
	Pakistan (n=25)		10 (40)			
	Jordan (n=16)		4 (25)			
	Libya (n=14)		3 (21)			
	Syria (n=12)		5 (42)			
	Sudan (n=12)		6 (50)			
	India (n=10)		5 (50)			
	Egypt (n=8)		5 (63)			
	Qatar (n=7)		3 (43)			
	United Kingdom (n=4)		1 (25)			
	Palestine (n=4)		2 (50)			
	Ireland (n=3)		1 (33)			
	Yemen (n=3)		1 (33)			
	United Arab Emirates (n=2)		1 (50)			
	Other unknown countries^a^ (n=38)		10 (26)			

^a^10 participants who scored ≥60% did not disclose their country of graduation.

## Discussion

Our study shows that postgraduate medical residents, fellows, and consultants in internal medicine or subspecialties at the largest tertiary care government hospital in Qatar have average to below-average knowledge in sleep medicine. The prevalence of obesity in Qatar is quite high, with 35% of men and 45% of women having a BMI higher than 30 [11]. Prevalence of obesity and consequent sleep-related breathing disorders are constantly rising, which can be attributed to the sedentary lifestyle, decreased physical activity, and unfavorable weather conditions possibly hindering a more active lifestyle. Sleep disorders are common worldwide; however, epidemiological studies on its prevalence are lacking in the State of Qatar. Anecdotal evidence reveals that, on average, 10 new patients with sleep-related breathing disorders are diagnosed in pulmonary clinics every week. This points to a high prevalence of sleep-related breathing disorders in the country.

In our study, only 35.8% of participants correctly answered more than 60% of the questions. Our results did not differ much from the previous studies assessing sleep knowledge across different countries in the Middle East region. For instance, a study comprising 215 physicians in Turkey showed that 45.3% of them answered questions correctly on a questionnaire assessing knowledge about sleep medicine [12]. Another similar study comprising primary health care physicians, of whom 94% were board-certified and 76% were certified in more than one field, rated their knowledge of sleep medicine as *fair* or *poor* [13]. In Egypt, Zaki et al [14] assessed the knowledge of normal sleep and sleep disorders among final-year medical students and house-officers from seven different medical faculties, also using the ASKME questionnaire. They found that 91% of the study participants had limited knowledge of sleep disorders, which is consistent with our results.

**Figure 2:**
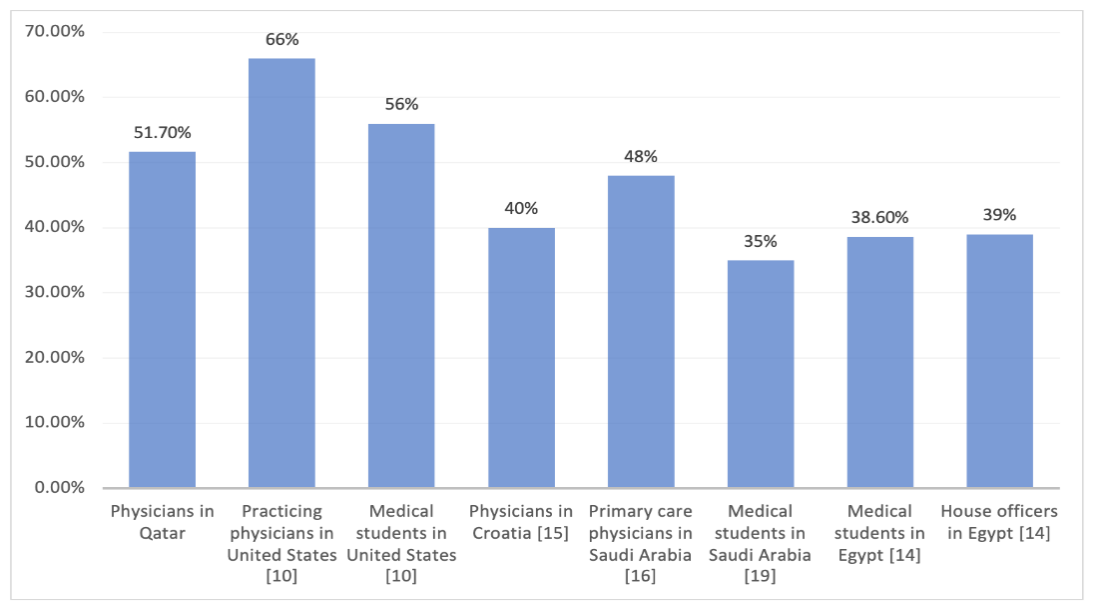
Comparison of the mean Assessment of Sleep Knowledge in Medical Education (ASKME) Survey scores obtained by physicians participating in this study and those obtained by physicians and students in studies in other countries using the same assessment tool [10,14,16,20].

In our study, the mean score obtained by the participants was 15.53 (ie, 51.7%), which was significantly lower than the mean score obtained by practicing physicians (66%) and medical students (56%) in the United states [10]. However, physicians in our study fared better than practicing physicians in Egypt, Croatia, and Saudi Arabia [14-16]. Comparison of mean ASKME scores of participants of our study and those of studies carried out in other countries is presented in Figure 2. Similar results were reported when a different survey was used to assess sleep knowledge. For example, a cross-sectional survey of general practice physicians in Ecuador, Peru, and Venezuela, using the OSA Knowledge and Attitudes (OSAKA) questionnaire demonstrated that although 73.5% of the physicians felt confident in identifying patients at risk for OSA, only 35.4% felt confident in managing these patients [17]. Similarly, behavior, attitude, and knowledge of sleep medicine assessed using MED sleep survey among interns and medical residents in university hospitals of central India revealed that average scores were 12.6 (ie, less than 50%). Moreover, 52.6% of the residents and 31.15% of the interns participating in this study obtained a score of more than 50%, which could be attributed to the increased exposure of residents to the medical literature [18]. Our study findings showed that trainees in fellowship programs had more knowledge in sleep than did interns and consultants, likely from increased exposure to consultations and a strong knowledge base from attending board certification residency exams.

It is worth mentioning an interesting study conducted in Hyderabad, India, wherein they found that only half of the practicing chest physicians could correctly answer 50% of the questions related to sleep-disordered breathing and only 10% of the respondents could answer 75% of the questions correctly [20]. These estimates demonstrate that even respiratory physicians exhibit a poor understanding of sleep disorders. Although our target population excluded chest physicians, our study shows similar results highlighting that after completion of graduate and/or postgraduate training, physicians are likely less exposed to updates or educational activities in sleep medicine, resulting in a decrement of knowledge of sleep disorders. Based on these findings, we can infer that health care providers worldwide exhibit a poor understanding of sleep disorders.

Most of the studies have not explored the obstacles related to poor knowledge of sleep disorders among health care providers. The lack of knowledge regarding sleep medicine could be the result of the limited time assigned for teaching sleep medicine at medical schools. A study from Saudi Arabia comprising undergraduate medical students using the ASKME Survey showed that the majority of the participants recorded their sleep knowledge as *below average*, with no difference in the scores observed among participants of different universities, gender, or academic level [19]. The main factor identified for this level of performance was the low priority for sleep medicine in the medical curriculum and the lack of time required to implement it. In 1998, a survey by the American Sleep Disorders Association and Sleep Research Society in the United States reported similar findings, with an average of 2.1 hours devoted to sleep medicine instruction at medical school. About 79% of respondents reported spending between 0.75 and 2.0 hours on the topic, 12% reported spending between 2.5 and 4 hours, and only 9% reported being provided 6-10 hours of sleep instruction. Respondents indicated that the greatest need was more instruction time—a tall order for an already crowded curriculum [21]. The absence of any significant difference in scores based on the country of graduation, as found in our study, also echoes these findings, thus underlining the deficiency of focus on sleep medicine in medical curricula across countries.

In the postgraduate internal medicine residency training program at our hospital, the average number of hours focused on sleep medicine training is less than 1 hour (in the form of didactic lectures). Majority of the knowledge is gained from clinical experience and through taking various international certification exams. Although there are faculty-trained and board-certified physicians in sleep medicine who regularly conduct sleep clinics with the support of a sleep laboratory run by two certified sleep technologists, there is no structured sleep medicine training or fellowship program in Qatar yet. This could also account for the low scores obtained among the trainees in our study. A review of sleep physiology and didactic lectures on obstructive sleep apnea and other sleep disorders along with their management is provided only in the pulmonary fellowship training program. Trainees in the pulmonary fellowship have electives in a sleep laboratory to understand how sleep studies are conducted and interpreted. Furthermore, a 2007 review of medical specialty textbooks found that information on sleep and sleep disorders constituted only about 2% of the overall content [22]. This lack of emphasis has contributed to the medical culture in which few physicians, other than sleep specialists, ask questions about sleep when recording a patient's history [8]. Our survey findings highlight the need for improving training in sleep medicine among postgraduate trainees in internal medicine and subspecialties. Didactic lectures can be complemented with sleep medicine modules. Introduction of educational modules has shown that successful learning can be achieved from these modules as well, when compared to the traditional educational metric on time spent on clinical rotation [23]. Distance learning and e-learning with collaborative institutes could also serve as a platform to enhance the knowledge and attitudes in sleep medicine. It is important to develop the structural framework for clinical experience, sleep education, conduct, and interpretation of sleep studies in relevant subspecialties. This would ensure more fellows entering the field of sleep medicine.

The gap between what we know about sleep and the limited exposure to that knowledge an average trainee or consultant receives at both the undergraduate and postgraduate levels highlights the need for more instruction time devoted to this topic. At the undergraduate level, integrating information on sleep and sleep disorders into the existing medical school curriculum could help, whereas at the postgraduate level, introduction of sleep modules and structured sleep medicine training programs may enhance knowledge of screening, diagnosis, and treatment of sleep disorders.

### Conclusions

Physicians working at Hamad Medical Corporation, Qatar, exhibit poor knowledge of sleep medicine, which could be attributed to the weak level of education in this field of medicine. Sleep disorders constitute a significant health problem and, if detected early, can generally be treated, improving the health and quality of life for these patients. Therefore, its necessary to emphasize on sleep medicine and sleep disorders during medical school education and residency training.

## Abbreviations

ACGME-I: The Accreditation Council for Graduate Medical Education–International
ASKME: Assessment of Sleep Knowledge in Medical Education
EDS: excessive daytime sleepiness
OSA: obstructive sleep apnea
OSAKA: Obstructive Sleep Apnea Knowledge and Attitudes
